# Morphology of the Antennal Sensilla of Notonectoidea and Comparison of Evolutionary Changes in Sensilla Types and Distribution in Infraorder Nepomorpha (Insecta: Heteroptera)

**DOI:** 10.3390/insects12121121

**Published:** 2021-12-14

**Authors:** Agnieszka Nowińska, Jolanta Brożek

**Affiliations:** Faculty of Natural Science, Institute of Biology, Biotechnology and Environmental Protection, University of Silesia in Katowice, Bankowa 9, 40-007 Katowice, Poland; agnieszka.nowinska@us.edu.pl

**Keywords:** sensory structures, Notonectidae, Pleidae, Helotrephidae, water bugs

## Abstract

**Simple Summary:**

Antennal sensilla are important sensory organs for insects. According to their morphological structures, they respond to different chemical or mechanical stimuli. The antennae of the studied families of water insects (Notonectidae, Pleidae and Helotrephidae) are short and concealed under the head, leaving a small amount of space for the existence of sensory structures. Nevertheless, six main types of sensilla have been discovered on the surfaces of these antennae. The morphological types described in this study were further compared with other studies on the antennal sensilla of water bugs (Nepomorpha) in order to compare their evolutionary changes within the group.

**Abstract:**

This article introduces the results of a study of three families of Nepomorpha and is the last part of a series of studies that sums up our work on the morphologies of the antennal sensory structures in this taxon. The morphologies and distribution of the sensilla in the families Notonectidae, Pleidae and Helotrephidae were studied under a scanning electron microscope. Six main types (sensilla trichodea, chaetica, campaniformia, basiconica, ampullacea and coeloconica) and ten subtypes (five subtypes of sensilla trichodea and five subtypes of sensilla basiconica) were described. The results were compared with other studies on the antennal sensilla of Nepomorpha in order to assess evolutionary changes within the infraorder. With the use of cladistics analysis, the monophyly of the families Nepidae, Micronectidae, Corixidae and Gelastocoridae was supported. On the other hand, the occurrence of some clades forming superfamilies was weakly supported by bootstrap analysis. These results, supported by presence of the numerous autapomorphies, suggest that antennal sensilla evolved within inner groups.

## 1. Introduction

Notonectoidea (Nepomorpha) form a monophyletic clade that includes the Notonectidae, Pleidae and Helotrephidae families [1,2,3,4,5,6,7]. Currently Ye et al. [8] and previously Štys and Jansson [9] recognized the superfamily Pleoidea (Pleidae + Helotrephidae) and Notonectoidea (Notonectidae). Based solely on phylogenetic (molecular) studies, Hua et al. [10] suggested placing Pleidae outside Nepomorpha, in a separate infraorder (Plemorpha). However, Weirauch and Schuh [5] considered this change in classification premature, noting a small taxon sample in the aforementioned analysis as well as the exclusion of the species’ morphology from the study. Notonectoidea (backswimmers) is a cosmopolitan taxon of aquatic insects that share the same method of transportation, swimming on their backs [11,12], but possess different behavioral strategies.

For the purpose of this study, we used specimens from all three families of Notonectoidea, as each has features specific to its own taxon such as different numbers of antennomeres and sets of antennal sensilla responsible for the recognition of mechanical and chemical signals from the water.

Notonectidae are small to medium sized (3.4–18 mm) water insects with two- to four-segmented antennae hidden beneath the eyes. They are generally predators which hunt by sight or by feeling vibrations on the water surface [11], their antennal mechanosensilla receiving stimuli from the water wave. Two predatory strategies were described by Gittelman [13]. *Notonecta* and *Buenoa* inhabit static waters where they sit and wait for prey. Upon noticing the prey, they dash suddenly to capture it. On the other hand, *Martarega* do not possess such capabilities of rapid acceleration. Instead, these insects swim against the current and try to spot prey stranded on the water surface within reach [13].

Helotrephidae are small (0.9–3.7 mm), strictly subaquatic insects with a round body shape. Their antennae are one- or two-segmented, although the sensilla types are not recognized easily. A unique phenomenon is the complete absence of antennae in brachypterous *Ficherotrephes*. Very little is known about Helotrephidae’s biology [11]. Two species have been studied more thoroughly, *Helotrephes semiglobosus formosanus* [14] and *Idiotrephes asiaticus* [15]. The Helotrephidae mostly inhabit lentic regions of running water, and can also be found in stagnant waters. While moving over larger distances, they swim venter up (like Pleidae and Notonectidae) or venter down, using the hind legs to row [14]. Flight has not been observed [11].

Pleidae are small (1.5–3.3 mm) insects with three-segmented antennae. The main difference between them and Notonectidae is that Pleidae have shorter rather than flattened hind legs. They typically inhabit stagnant waters with rich vegetation, where they can hide. They are good swimmers, despite their short hind legs. Pleidae are predators like Notonectidae and Helotrephidae, feeding on small invertebrates and even younger instars of their own species [11,16].

Antennae play a crucial role in the life of insects [17], as they receive stimuli related to food location, nest location, inter and intra-specific recognition, mating and the suitability of environmental conditions [18]. Specialized receptors that form part of the antennal epidermis (antennal sensilla) comprise the link between stimuli and behaviour. The matter of interest in this study is the antennal sensilla, sensory structures which display functions of mechano-, chemo-, and thermo-hygroreception. Such structures are composed of auxiliary cells—modified epidermal cells—and sensory neurons surrounded by a dendrite sheath. The signal is captured by a specialized sensor in the cuticle and converted to be carried by sensory neurons to the brain. The type of a sensillum can be determined based on its external structure. Usually, sensilla with a porous surface are chemoreceptive sensilla. Mechano- and thermo-hygrosensilla do not have pores [19,20]. Another important morphological character of sensilla is the socket; flexible sockets are characteristic of mechnosensilla and contact chemoreceptive sensilla (bimodal), whereas inflexible sockets are specific to chemosensilla and thermo-hygrosensilla [21].

Apart from morphological variations among sensilla types, there is also an important variability in the incidence, density, and distribution of the different types of sensilla among different insect species (e.g., Hymenoptera) even within a single genus, and between sexes within a species [22,23]. Similar variations in types, density and distribution of sensilla have also been observed in the studied taxa of the nepomorphan species [24,25,26,27]; however, their phylogenetic effects are still little explored. In addition to the putative phylogenetic effects on such variability (as in Hymenoptera’s species, for instance [28]), the diversity, density, and distribution patterns of sensilla may be the product of interacting selection pressures related to feeding and foraging habits, habitat type, mating systems [29,30,31] and the insects’ systematic position.

The basic morphology of the antennal sensilla as well as the ultrastructure of the receptors of several taxa of the infraorder Nepomorpha were studied by Chaika and Sinitsina [32]. More detailed studies on the morphologies, distributions and functions of antennal sensilla were conducted in a systemic approach, based on numerous species of Nepidae, Belostomatidae [24], Aphelocheiridae, Ochteridae, Gelastocoridae [25], Corixidae, Micronectidae [26] and Naucoridae [27]. Moreover, Garza et al. [33] published research on the antennae and sensilla of some species of Pleidae, with an emphasis on their morphologies and suspected functions. The present study on Notonectoidean taxa and aforementioned water bugs allows for a detailed comparison of antennal morphology and sensillar equipment between many families, subfamilies and species with the goal of proposing preliminary hypotheses on their evolution, by mapping salient traits and comparing them with the most recent nepomorphan phylogeny presented by Ye et al. [8].

The goal of this study is to describe and analyze the morphologies and functions of the antennal sensillar system of Notonectoidea (Notonectidae, Pleidae and Helotrephidae), taking into account evolutionary changes in particular families. Moreover, the study aims to provide more general insight into the different antennal sensilla of the nepomorphan bugs, and attempts to clarify whether the different types of sensilla and their distribution have a phylogenetic value based on the studied taxa. Two hypotheses are proposed regarding the differentiation of sensilla in individual taxa of the water bugs:

- The sensilla type and their arrangement on the antennae have a phylogenetic value (synapomorphies) resulting from the relationships of the taxa.
- The sensilla type and their arrangement on the antennae are the result of their evolution in the aquatic environment.

## 2. Materials and Methods

### 2.1. Taxon Samples

**Figure 1:**
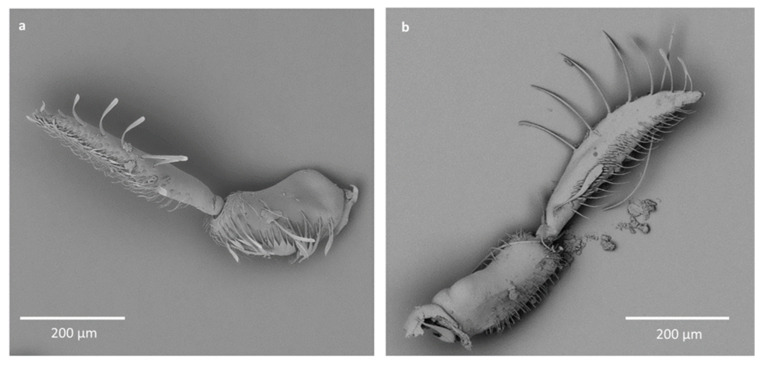
The antennae of Anisopinae; (**a**)—*Anisops sardea* female, (**b**)—*Anisops sardea* male.

Materials were obtained thanks to a personal donation from Dr. Ping-Ping Chen, as well as from the collections of the Natural History Museum in Vienna, the Moravian Museum in Brno and the Hungarian Natural History Museum in Budapest. At least three specimens of each species were used for the study. Most of the specimens were female; however, male and female representatives of the species *Anisops sardea* were examined (Figure 1). No significant differences were observed, and therefore, the sex of the specimens was generally not taken into consideration. All specimens were cleaned in an ultrasound cleaner (Polsonic, Warsaw, Poland), the antennae were dissected, dried in ethanol, mounted, sputtered with gold or chromium with the use of the turbomolecular pump coater (Quorum 150T ES plus—Quorum Technologies, Laughton, East Sussex, UK), and both sides of the antennae were observed with the use of the scanning electron microscopes (Phenom XL Phenom-World BV, Eindhoven, The Netherlands and Hitachi UHR FE-SEM SU 8010 High Technologies, Tokyo, Japan) in the scanning microscopy laboratory of the Faculty of Natural Science, Institute of Biology, Biotechnology and Environmental Protection of Silesian University in Katowice. We followed the terminology and classification reported in other papers on the antennal sensilla of insects [19,21,34,35].

The antennae of sixteen species from three families were studied:
Notonectidae:Anisopinae:
*Anisops debilis* Gerstaecker, 1873


*Anisops jaczewski* Hutchinson, 1928


*Anisops sardea* Herrich-Schaeffer, 1850


*Buenoa nitida* Truxal, 1953
Notonectinae:Notonectini:*Enithares metallica* Brooks, 1948


*Enithares stridulata* Brooks, 1948


*Notonecta ceres ceres* Kirkaldy, 1897


*Notonecta disturbata* Hungerford, 1926


*Notonecta glauca* Linnaeus, 1758


*Notonecta maculata* Fabricius, 1794

Nychiini:*Martarega gonostyla* Truxal, 1949


*Martarega uruguayensis* Berg, 1883Pleidae:

*Plea minutissima* Leach, 1818


*Paraplea* sp.Helotrephidae:Helotrephinae:
*Hydrotrephes visayasensis* Zettel, 2003
Neotrephinae:
*Neotrephes lanemeloi* Nieser and Chen, 2002

### 2.2. Programs Used for Cladistic Analysis

In the results and discussion section of this paper, the morphology and the preliminary estimation of the characters of the antennal sensilla (in respect of phylogenetic value) of the outgroup Gerromorpha and the Nepoidea taxon (Nepidae and Belostomatidae) are compared with the more diverse forms of these structures in more evolutionarily advanced groups (i.e., Ochteridae, Gelastocoridae, Aphelocheiridae, Corixidae, Naucoridae and Notonectoidea (Pleidae, Helotrephidae and Notonectidae).

These sensilla have been analyzed regarding evolutionary aspects based on the currently studied taxon (Notonectoidea) and the previously available data on Nepomorpha, after Nowińska and Brożek [24,25,27], Nowińska et al. [26] and data on Gerromorpha, after Nowińska and Brożek [35]. The morphological cladistics analysis of the antennal sensilla of Nepomorpha was performed on a phylogenetic program of Tree Analysis Technology TNT and Winclada. The dataset included the studied taxon’s sampling data, and all morphological characteristics were treated as ordered. Matrix characters (Table 1) were set in several states, showing the types and distributions of the sensilla according to their presence/absence in the particular systematic position of the taxa based on the phylogenetic tree of Ye et al. [8]. For the maximum parsimony (MP) analyses, heuristic searches were performed with TNT version 1.6 [36], using traditional search algorithms. Additionally, branches of the tree were supported by a Bootstrap analysis. Exploration of the effects of homoplasy on the results of implied weighting (IW) was also performed for the “all taxa” category, with constants of concavity (k) set to different integer values between 3 and 15, separated by 1 integer. It has been demonstrated that, when properly done, weighting characteristics according to their homoplasy produces more strongly supported groups (k = 12) and more stable results in analyses of morphological datasets [36]. The tree with the highest mean similarity was chosen as the working hypothesis tree in order to optimize the characteristics (L 342, Ci 28, Ri 63). The unambig and slow optimization changes were mapped on the tree using Winclada [37]. Non-homoplasy style is presented in a green box and a homoplasy style is marked as a red circle. The selected MP tree shows a slow optimization of morphological characters with unique and homoplastic changes in a red circle.

### 2.3. Description of the Character States

The matrix above was prepared based on the number of antennomeres, the types of sensilla and their distribution on individual antennomeres. Species whose antennomeres were destroyed during dissection were excluded from the list.

1. Number of antennomeres: (0) 4, 5 (1) 3, (2) 2, (3) 1,
2. Sensilla trichodea ST1: (0) present on the 3rd and 4th antennomeres, (1) present on all antennomeres or absent only on the first, (2) absent on all antennomeres (3) absent only on the 3rd antennomere (4) present only on the 3rd antennomere, (5) present on the 2nd antennomere or on the 2nd and 3nd antennomeres, (6) absent only on the 4th antennomere, (7) absent only on the 2nd antennomere, (8) present only on the 4th antennomere (9) present only on the 1st and 2nd antennomeres,
3. Sensilla trichodea ST2: (0) present on all antennomeres, (1) absent on all antennomeres, (2) absent only on the 1st antennomere, (3) present only on the 3rd antennomere, (4) present only on the 1st antennomere, (5) present only on the 2nd antennomere, (6) present only on the 1st and 2nd antennomeres, (7) absent only on the 2nd antennomere, (8) present only on the 2nd and 3rd antennomeres, (9) present only on the 4th antennomere,
4. Sensilla trichodea ST3: (0) present on the 1st and 2nd antennomeres (1) present only on the 3rd and 4th antennomeres, (2) absent, (3) present only on the 3rd antennomere, (4) present only on the 1st antennomere (5) present only on the 2nd antennomere, (6) present only on the 2nd and 4th antennomeres,
5. Sensilla trichodea ST4a: (0) absent, (1) present only on the 3rd antennomere,
6. Sensilla trichodea ST4b: (0) absent, (1) absent only on the 1st antennomere, (2) present only on the 3rd and 4th antennomeres, (3) present only on the 3rd antennomere,
7. Sensilla trichodea ST4c: (0) absent, (1) present only on the 3rd antennomere, (2) present only on the 3rd and 4th antennomeres, (3) present only on the 2nd antennomere,
8. Sensilla trichodea ST5a: (0) absent, (1) present only on the 3rd antennomere, (2) present only on the 3rd and 4th antennomeres,
9. Sensilla trichodea ST5b: (0) absent, (1) present only on the 1st antennomere, (2) present on the 2nd antennomere, (3) present only on the 3rd antennomere, (4) present only on the 2nd and 3rd antennomeres, (6) absent only on the 1st antennomere, (6) present only on the 3rd and 4th antennomeres, (7) present only on the 2nd and 4th antennomeres,
10. Sensilla chaetica SCh: (0) present on all antennomeres, (1) absent, (2) present only on the 3rd and 4th antennomeres, (3) present only on the 4th antennomere, (4) present only on the 2nd antennomere, (5) present only on the 1st antennomere,
11. Sensilla cone-like SCoL: (0) absent, (1) present,
12. Sensilla brush-like SBL: (0) absent, (1) present,
13. Sensilla club-like SClL: (0) absent, (1) present only on the 1st antennomere, (2) present only on the 1st and 2nd antennomeres,
14. Sensilla paddle-like SPL1: (0) absent, (1) present only on the 1st antennomere, (2) present only on the 3rd antennomere,
15. Sensilla paddle-like SPL2: (0) absent, (1) present,
16. Sensilla squamiformia SSq: (0) absent, (1) present,
17. Sensilla campaniformia SCa: (0) present, (1) absent,
18. Sensilla basiconica SB1: (0) present, (1) absent,
19. Sensilla basiconica SB2: (0) present only on the 4th antennomere, (1) present only on the 3rd and 4th antennomeres, (2) present only on the 2nd and 3rd antennomeres (3) absent, (4) present on the 2nd, 3rd and 4th antennomeres, (5) present only on the 1st antennomere, (6) present only on the 3rd antennomere, (7) absent only on the 1st antennomere,
20. Sensilla basiconica SB3a: (0) absent, (1) present only on the 3rd and 4th antennomeres, (2) present only on the 4th antennomere, (3) present only on the 3rd antennomere, (4) present only on the 2nd and 3rd antennomere, (5) present only on the 2nd antennomere,
21. Sensilla basiconica SB3b: (0) absent, (1) present only on the 2nd antennomere, (2) present only on the 2nd and 3rd antennomeres, (3) present only on the 3rd antennomere, (4) present only on the 3rd and 4th antennomeres, (5) present only on the 4th antennomere,
22. Sensilla basiconica SB3c: (0) absent, (1) present only on the 3rd and 4th antennomeres, (2) present on the 2nd and 3rd antennomeres,
23. Sensilla basiconica SB4: (0) present only on the 4th antennomere, (1) absent, (2) present only on the 3rd antennomere, (3) present only on the 2nd, 3rd and 4th antennomeres, (4) present only on the 2nd and 3rd antennomeres, (5) present only on the 2nd and 4th antennomeres, (6) present only on the 2nd antennomere, (7) present only on the 3rd and 4th antennomeres,
24. Sensilla coeloconica SCo1a: (0) absent, (1) present,
25. Sensilla coeloconica SCo2a: (0) absent, (1) present only on the 4th antennomere, (2) present on the 3rd antennomere,
26. Sensilla coeloconica SCo3: (0) absent, (1) present only on the 3rd and 4th antennomeres, (2) present only on the 2nd and 3rd antennomeres,
27. Sensilla coeloconica SCo1b: (0) sparsely distributed all over the antenna, (1) absent, (2) present only on the 3rd antennomere, (3) present only on the 3rd and 4th antennomeres, (4) present only on the 2nd antennomere, (5) present only on the 4th antennomere (6) present only on the 2nd and 3rd antennomeres,
28. Sensilla coeloconica SCo2b: (0) absent, (1) present on the 3rd antennomere, (2) present on the 4th antennomere,
29. Sensilla ampullacea SA: (0) present, (1) absent,
30. Sensilla plate-like SPl: (0) absent, (1) present,
31. Sensilla placodea multilobated SPM: (0) absent, (1) present,
32. Sensilla trichodea curved: (0) present, (1) absent.

## 3. Results

**Figure 2:**
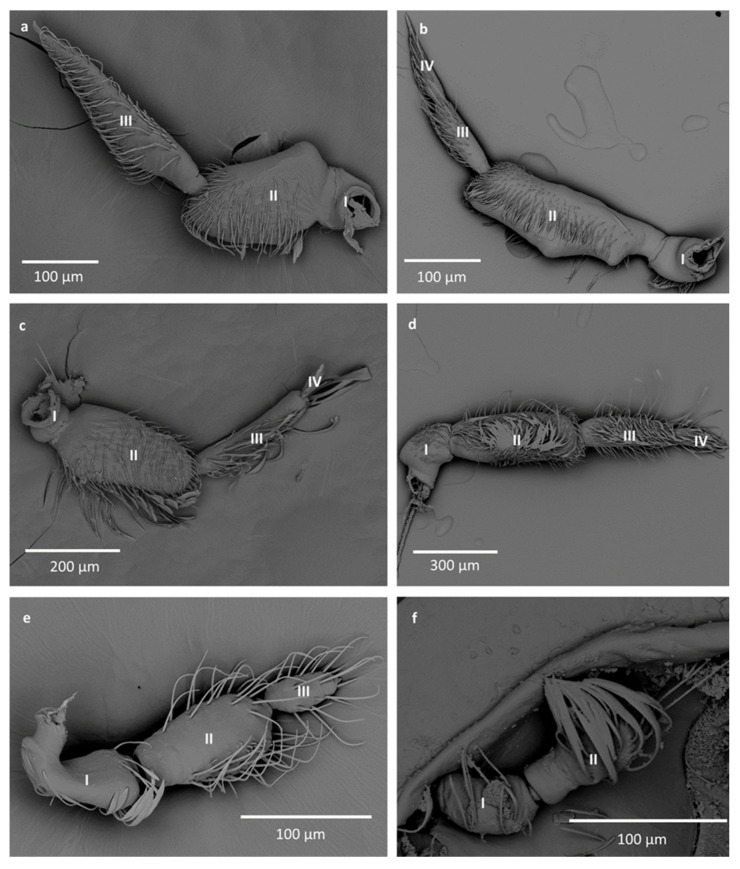
The antennae of studied species; (**a**)—*Anisops jaczewski*, (**b**)—*Enithrates stridulata*, (**c**)—*Martarega uruguayensis*, (**d**)—*Notonecta ceres ceres*, (**e**)—*Paraplea* sp., (**f**)—*Hydrotrephes visayasensis*. The numbers I, II, III, IV correspond to the segments of the antennae.

Species with 1–4 antennomeres were studied. The shapes of the antennae differ among the species due to different segmentation; however, some similarities are present. The two first segments are larger than the rest of the antenna, with the second one usually being the largest, and the rest is visibly thinner (Figure 2). During dissection, some very fine or very small antennomeres were destroyed. To compensate, whole antennae of other species from the same genera are presented.

In Anisopinae, the antennae consist of three antennomeres. The first is the shortest, the second is the widest, and the third is long and thin (Figure 1 and Figure 2a).

In Notonectinae, three different shapes of antennae were observed; all had four segments. In *Enithares* the first antennomere is the shortest, the second is long and wide with small protuberances, and the last two antennomeres are very thin and almost the same length (Figure 2b). In *Martarega*, the first antennomere is short, the second is the widest and is much longer than the first, the third is long and thin, and the last antennomere is very short and thin (Figure 2c). In *Notonecta*, the first and last antennomeres are short, while the second and third are long. The second antennomere is also the widest (Figure 2d).

In Pleidae, the antennae are three-segmented. The first and second are longer and wider, and the third is the shortest and the smallest (Figure 2e).

In Helothrephidae, only one- and two-segmented antennae were observed. In *Neotrephes,* the single antennomere is oval shaped, with similar width and length. In *Hydrotrephes*, the first and second antennomeres have similar length and width (Figure 2f).

### 3.1. Morphology and Categories of Antennal Sensilla

**Figure 3:**
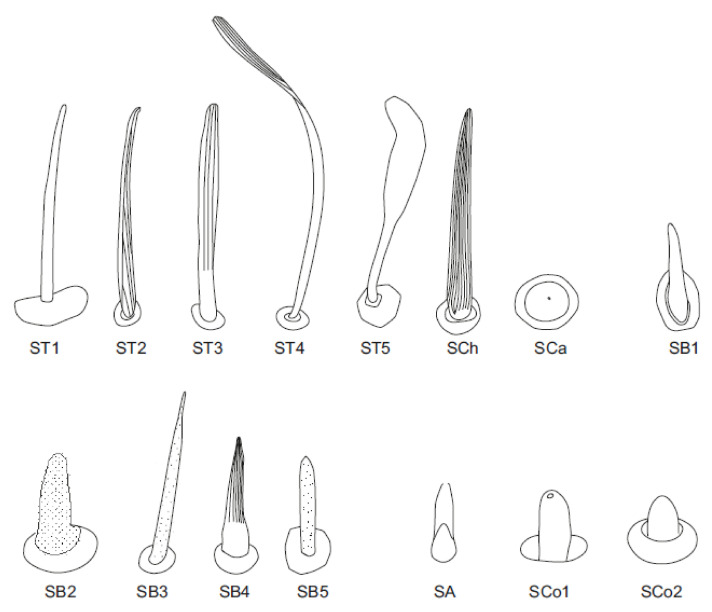
Schematics of the types of sensilla in the studied species. ST—sensilla trichodea; SCh—sensilla chaetica; SCa—sensilla campaniformia; SB—sensilla basiconica; SA—sensilla ampullacea; SCo—sensilla coeloconica.

**Figure 4:**
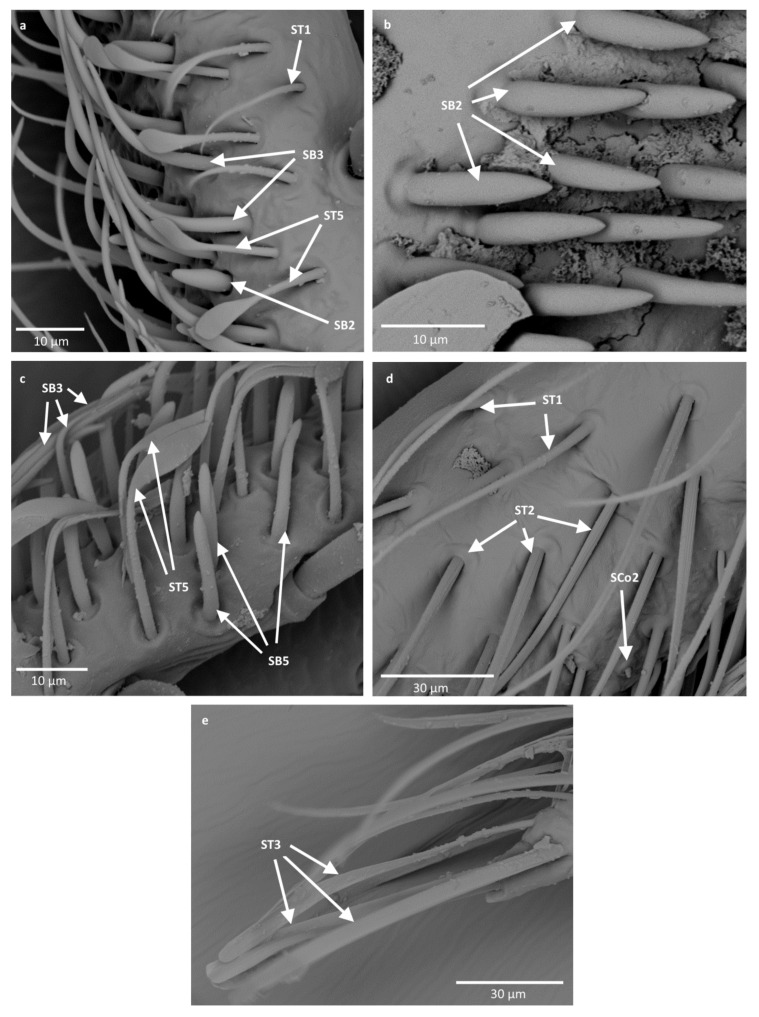
The surface of the antennae of Notonectidae; (**a**)—*Anisops debilis*, (**b**)—*Anisops sardea*, (**c**)—*Buenoa nitida*, (**d**)—*Enithrates stridulata*, (**e**)—*Enithrates metalica*. ST—sensilla trichodea; SB—sensilla basiconica; SCo—sensilla coeloconica.

**Figure 5:**
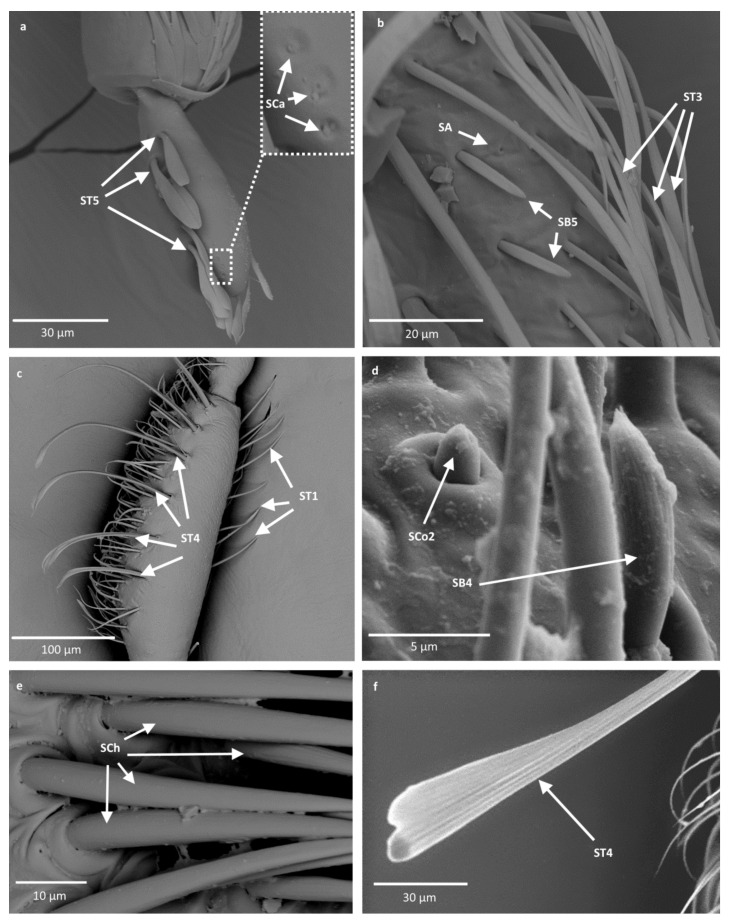
The surface of the antennae of Notonectidae; (**a**,**b**)—*Martarega uruguayensis*, (**c**)—*Notonecta disturbata*, (**d**)—*Notonecta glauca*, (**e**)—*Notonecta ceres ceres*, (**f**)—*Notonecta maculata*. ST—sensilla trichodea; Sca—sensilla campaniformia; SCh—sensilla chaetica; SB—sensilla basiconica; SCo—sensilla coeloconica; SA—sensilla ampullacea.

**Figure 6:**
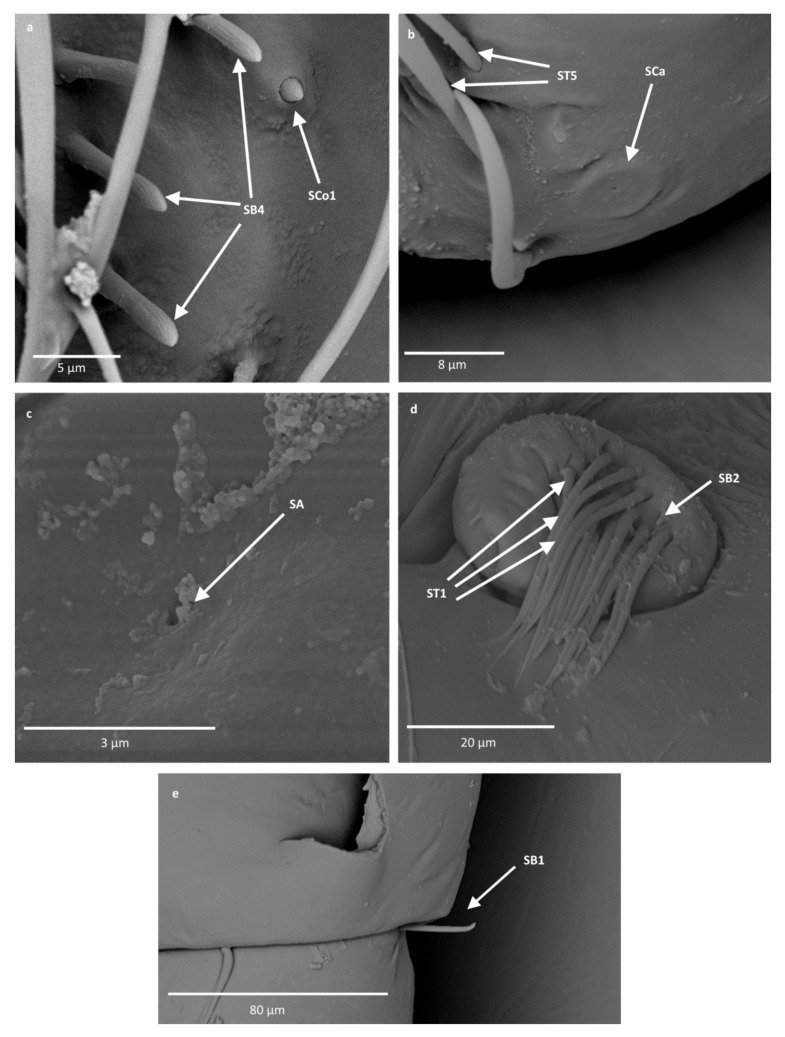
The surface of the antennae of Pleidae and Helotrephidae; (**a**)—*Plea minutissima*, (**b**)—*Paraplea* sp., (**c**)—*Hydrotrephes visayasensis*, (**d**)—*Neotrephes lanemeloi*, (**e**)—*Notonecta ceres ceres*. ST—sensilla trichodea; Sca—sensilla campaniformia; SB—sensilla basiconica; SCo—sensilla coeloconica; SA—sensilla ampullacea.

The external morphological characters indicating the types of sensilla are the pores (visible or not), the manner in which the sensilla are embedded with respect to the surface of the antennae (flexible or inflexible sockets), and the shape of the sensilla. All recognized shapes of sensilla are presented in Figure 3 and their locations in Notonectidae, Pleidae and Helotrephidae are presented in Table 2. Functionally, the antennal sensilla are classified into three categories: mechanoreceptive, chemoreceptive and thermo-hygroreceptive sensilla. The following is a description of the various sensilla types and subtypes:

1. Sensilla trichodea—sensilla responsible for mechanoreception. Straight, long and hair like structures. Usually, they cover a large surface on the antennae. They arise from flexible sockets, which is common for mechanoreceptive sensilla. A flexible socket is a socket with a thin cuticular membrane that is continuous with the cuticle and hair of the general body. A flexible socket provides greater mobility at the base of the sensillum. The following sensilla trichodea subtypes were observed:
  - Sensilla trichodea (ST1)—straight, hair-like structures with a smooth surface and pointed tip. These sensilla were found in large numbers on different antennomeres in all studied species except for *Neotrephes lanemeloi* (Figure 3, Figure 4a,d, Figure 5c and Figure 6d).
  - Sensilla trichodea (ST2)—straight structures with a pointed tip and ribbed surface. This subtype occurs often on insect antennae, and was found among sensilla trichodea ST1. They were found in all studied species except *Paraplea* and Helotrephidae species (Figure 3 and Figure 4d).
  - Sensilla trichodea (ST3)—long structures with a ribbed surface on one side, widening more less in the middle. These sensilla were usually found on the second last and last antennomeres. They usually cover a large part of the antennomere, protecting smaller sensilla underneath. They were found in *Anisops debilis*, *Anisops sardea*, *Buenoa nitida*, *Enithares*, *Martarega uruguayensis* and *Hydrotrephes visayasensis*, on different antennomeres (Figure 3, Figure 4e and Figure 5b).
  - Sensilla trichodea (ST4)—long structures protruding above all other sensilla. They are ribbed and resemble an oar. These sensilla were present in all studied species, except *Enithares* and the species of Pleidae and Helotrephidae. They were found on the second last and last antennomeres, usually organized in a row through the length of the antennomere (Figure 3 and Figure 5c,f).
  - Sensilla trichodea (ST5)—long structures, cylindrical at the base with the rest of sensillum flattened and resembling a leaf. Overall, they were found on every antennomere. Depending on the species, however, they occur on one, two or three antennomeres, but never in all four of them at once. As with sensilla trichodea ST3, they cover a large part of the antennomere, protecting the smaller sensilla underneath. They were found on different antennomeres in all species except *Enithares* and *Neotrephes lanemeloi* (Figure 3, Figure 4a,c, Figure 5a and Figure 6a).
2. Sensilla chaetica (SCh)—long structures, much more rigid than trichoid sensilla. They are sharpened at the tip, ribbed, and arise from flexible sockets. Sensilla chaetica are much less common than sensilla trichodea. They were only found in *Notonecta*, grouped on the second antennomere, except in *Notonecta disturbata* (we expect that sensilla chaetica are also present there; however, the antennae of this species were on a slightly different angle and therefore it is possible that the sensilla were occluded from view) (Figure 3 and Figure 5e).
3. Sensilla campaniformia (SCa)—sensilla also responsible for mechanoreception, therefore arising from flexible sockets. These are flat structures, resembling round or oval discs, with a single pore in the middle. This type was found on different antennomeres in *Buenoa nitida*, *Enithares stridulata*, *Notonecta ceres ceres*, *Martega*, *Paraplea* sp. and in the species of Helotrephidae (Figure 3, Figure 5a and Figure 6b).
4. Sensilla basiconica (SB)—sensilla responsible for chemoreception in general, usually olfaction. Their surface is covered with wall pores which allow chemical particles to enter. Unlike mechanoreceptive sensilla, these structures arise from inflexible sockets. One subtype was found which we believe to be responsible for proprioception. The following subtypes were observed:
  - Sensilla basiconica (SB1)—long, smooth structures occurring singularly between antennomeres. These sensilla have a proprioceptive function. They were found in *Enithares*, *Notonecta ceres ceres*, *Notonecta disturbata* and *Paraplea* sp. on the first antennomere (Figure 3 and Figure 6e).
  - Sensilla basiconica (SB2)—cone-like structures with a porous surface and slightly pointed tip. They were found in all studied species, except *Buenoa nitida*, *Enithares metallica*, *Notonecta disturbata*, *Martarega*, *Plea minutissima*, *Hydrotrephes visayasensis* and *Neotrephes lanemeloi*. This type was found on different antennomeres, both singularly and in groups (Figure 3, Figure 4a,b and Figure 6d).
  - Sensilla basiconica (SB3)—long, porous structures with a thick base and a thinner, slightly pointed tip. They were found in all studied species except *Martarega* and in the species of Helotrephidae. This type generally occurred on the second last and last antennomeres, in a group among sensilla trichodea and other sensilla basiconica. It is unusual for sensilla basiconica to be this long, but their external structure suggests a chemosensory function (Figure 3 and Figure 4a,c).
  - Sensilla basiconica (SB4)—cone-like structures, smooth at the base and ribbed along the length of sensillum. They were found in *Anisops debilis*, *Anisops jaczewski*, *Enithares*, *Notonecta ceres ceres* and *Plea minutissima*. These sensilla were found among sensilla trichodea ST3 and ST5, which covered and protected them (Figure 3, Figure 5d and Figure 6a).
  - Sensilla basiconica (SB5)—structures resembling flattened cones. They are more or less the same length as sensilla basiconica, SB2 and SB3, with a round base that flattens along the rest of the sensillum. Their tip is rounded or slightly rounded. They were found in *Buenoa nitida*, *Enithares*, *Notonecta ceres ceres*, *Notonecta disturbata*, *Martarega* and *Plea minutissima*. These sensilla were found in groups; mostly they appeared as single structures on different antennomeres (Figure 3, Figure 4c and Figure 5b).
5. Sensilla coeloconica (SCo)—sensilla responsible for thermo-hygroreception. They are peg-like structures embedded in a cavity of cuticle in inflexible sockets. The following subtypes were observed:
  - Sensilla coeloconica (SCo1)—pegs arising from the cuticle, sometimes on a protuberance of the cuticle. They were found on the top of the antennae in *Notonecta ceres ceres*, *Notonecta glauca* and *Martarega*. These sensilla were also present on the surface of the last two antennomeres in *Plea minutissima* and *Paraplea* (Figure 3 and Figure 6a).
  Sensilla coeloconica (SCo2)—pegs arising from the cuticle, surrounded by a cuticular shaft. They were found on the surface of the second last and last antennomere, covered by sensilla trichodea, ST3 and ST5, in all studied species of Notonectidae, except in *Anisops sardea*, *Buenoa nitida* and *Martarega gonostyla* (Figure 3, Figure 4d and Figure 5d).
6. Sensilla ampullacea (SA)—sensilla responsible for thermo-hygroreception. They are small pegs, hidden in a deep cavity and arising from inflexible sockets. They occurred singularly on different antennomeres in a few of the studied species. Only an opening to the cavity is visible from the outside, and therefore we are not able to confirm that those are, in fact, sensilla ampullacea. However, taking into account this morphological appearance and comparing it with other possibilities (such as glandular structures, which usually form a sieve structure rather than appear singularly), we believe these to be sensilla ampullacea. These structures were found in *Anisops sardea*, *Notonecta ceres ceres*, *Martarega uruguayensis* and *Hydrotrephes visayasensis* (Figure 3, Figure 5b and Figure 6c).

**Table 2:** Distribution of sensilla types on the antennomeres of the studied species.

Species Name	Sensilla Present on the I Antennomere	Sensilla Present on the I Antennomere	Sensilla Present on the III Antennomere	Sensilla Present on the III Antennomere
*Anisops debilis*	antennomere destroyed during dissection	ST1, ST2, ST3, ST4	ST4, ST5, SB2, SB3, SB4, SCo2	-
*Anisops jaczewski*	ST1, ST2	ST1, ST2, ST5	ST1, ST2, ST4, ST5, SCa, SB2, SB3, SB4, SCo2	-
*Anisops sardea*	antennomere destroyed during dissection	ST1, ST2, ST3, ST5, SA	ST4, ST5, SB2, SB3	-
*Buenoa nitida*	ST1, ST2,	ST1, ST2, ST3, SCa	ST4, ST5, SB3, SB5	-
*Enithares metallica*	ST1, ST2, SB1	ST1, ST2, ST3, SB5	ST1, ST2, SB3, SB5	ST2, ST3, SB3, SB4, SCo2
*Enithares stridulata*	ST1, ST2, SCa, SB1	ST3, SB5	ST1, ST2, SB3, SB4, SCo2	ST1, ST2, ST3, SB2
*Notonecta ceres ceres*	SCa, SA, SB1	ST1, ST2, ST5, SCh, SA	ST1, ST2, ST5, ST4, SB2, SB3, SB4, SCo2	ST1, ST2, ST4, ST5, SB2, SB5, SCo1
*Notonecta disturbata*	ST1, ST2, SB1	ST1, ST2, ST5	ST1, ST2, ST4, ST5, SCa, SB3, SCo2	ST1, ST2, ST4, ST5, SB5
*Notonecta glauca*	ST1, ST2	ST1, ST2, ST5, SCh	ST1, ST2, ST4, SB2, SB3, SCo2	ST1, ST2, ST4, ST5, SB2, SB3, SCo1
*Notonecta maculata*	ST1, ST2	ST1, ST2, ST5, SCh	ST1, ST2, ST4, ST5, SB2, SCo2	ST1, ST2, ST5, SB2, SB3
*Martarega gonostyla*	SCa	ST1, ST2, ST5	ST1, ST2, ST4, ST5, SCa, SB5, SCo1	antennomere destroyed during dissection
*Martarega uruguayensis*	SCa	ST1, ST2, ST4, ST5, SCa	ST1, ST2, ST3, ST4, SB5, SA, SCo2	ST5, SCa, SCo1
*Plea minutissima*	ST1, ST2, ST5	ST1, SB3, SB4, SCo1	ST1, SB5, SCo1	-
*Paraplea* sp.	ST5, SCa, SB1	ST1, SB2, SB3, SB4, SCo1	ST1, SB2, SB3, SCo1	-
*Hydrotrephes visayasensis*	ST1, ST3, SCa, SA	ST1, ST2, ST5, SCa, SB2	-	-
*Neotrephes lanemeloi*	ST1, SCa, SB2	-	-	-

### 3.2. Cladistic Analysis of the Morphological Characteristics of Antennal Sensilla in Nepomorpha

**Figure 7:**
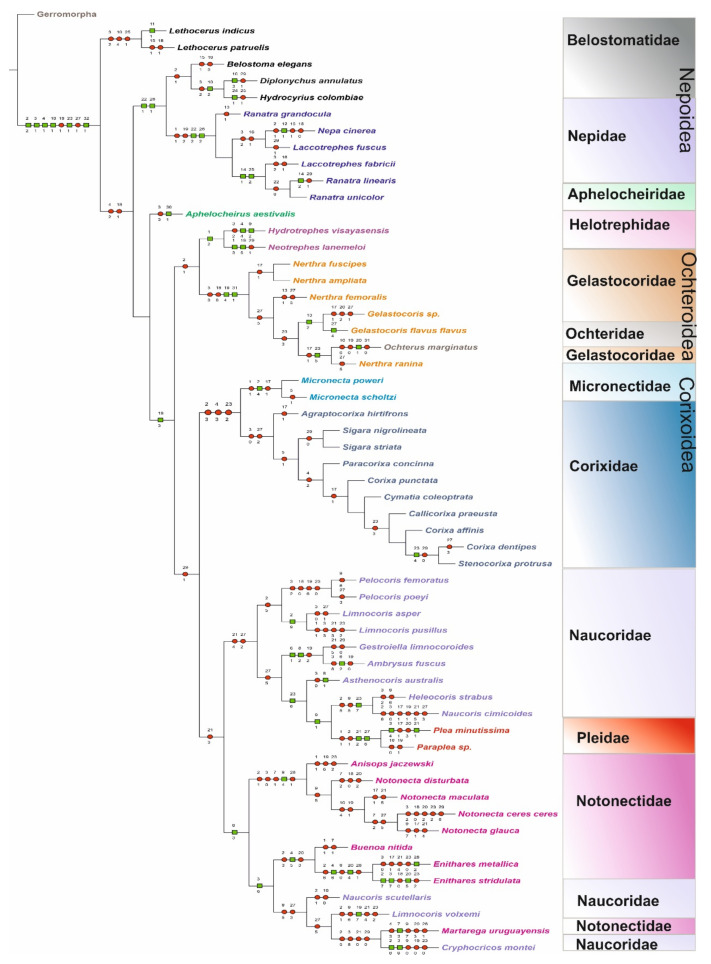
The MP tree resulting from the traditional search, with characters treated as ordered and implied weighting in TNT, as visualized in Winclada. A small green box indicates non-homoplasy (synapomorphies and autapomorphies); a red small box indicates homoplasy. The number above the branch line refers to the number of a character; the number below the line of the branch refers to the number of the state of a character.

**Figure 8:**
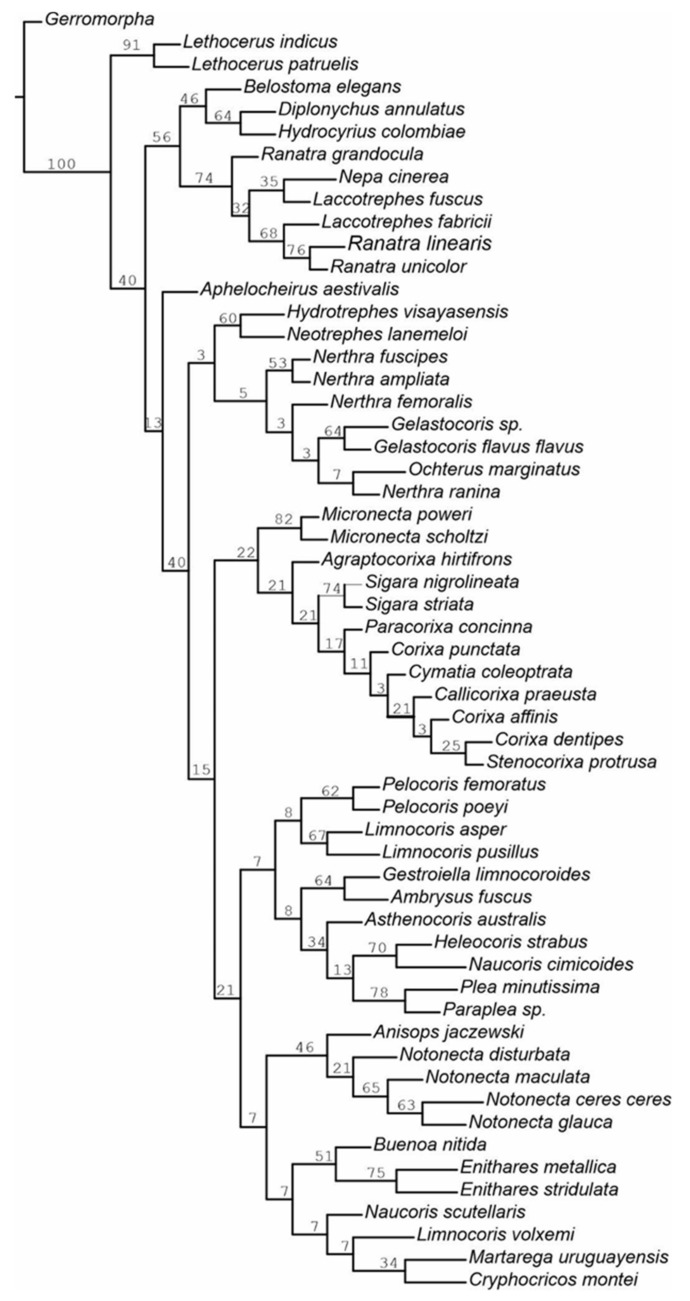
Bootstrap support; an MP tree of the character dataset showing the bootstrap support for clades.

The heuristic search strategy yielded 43 parsimonious trees, 342–351 steps long and with a consistency index of 28 and a retention index of 63. The shortest tree (342 steps long, with the complete mapping of all morphological characters as non-homoplasious = syn(apomorphies) and homoplasious) (Figure 7) represents the hypothesis concerning the relationship between the nepomorphan families given below. The bootstrap analysis of morphological characters is also shown in Figure 8.

Based on the cladistics analysis, synapomorphic and autapomorphic characters were distinguished for the taxa of the infraorder in slow optimization (Figure 7).

In the MP tree (Figure 7), the first step leads to the upper branch of the superfamily of Nepoidea, and the lower branch represents the remaining taxa. The Nepoidea (Nepidae and some Belostomatidae) are recognized on the basis of two synapomorphies (22-1), (26-1). However, the two species of Lethocerinae are posted separately via homoplastic characters. *Lethocerus indicus* is distinguished with one autapomorphy (11-1). *Diplonychus* and *Hydrocyrius* are grouped by synapomorphy (10-2). Each of the species has one autapomorphy (10-2 and 24-1). The family Nepidae represents two synapomorphies (22-2, 26-2). Inside the family, one clade (*Laccotrephes fabricii*, *Ranatra linearis* and *Ranatra unicolor)* with two synapomorphies (14-1 and 25-2) has been distinguished. The Belostomatidae are a sister group to Nepidae.

The next branch represents *Aphelocheirus aestivalis*, with a strong autapomorphy (30-1). The position of *Aphelocheirus* is the result of the character (2-2) in common with the Lethocerinae *Laccotrephes* and *Ranatra* (absence of the ST1), which indicates a parallel character.

Helotrephidae is supported by one synapomorphy (1-2); It is a unique character only for *Hydrotrephes* (two segmented antennae), however. Both species have autapomorphy (*Hydrotrephes visayasensis* 4-4 and 9-2; *Neotrephes lanemeloi* 1-3 and 19-5). Helotrephidae appears as a sister clade to Ochteroidea.

There are two synapomorphies (19-4 and 31-1) for three species of Nerthrinae. In the case of the Gelastocorinae, one synapomorphy (13-2) is visible. Gelastocorinae are a sister group to *Ochterus marginatus* and *Nerthra ranina.* This clade is distinguished by synapomorphy (23-6). *Ochterus* possesses one autapomorphy (20-1) that has given support to the family level of Ochteridae. *Ochterus* belongs to a branch together with Gelastocoridae. These characters probably support a monophyletic status of Ochteroidea (Gelastocoridae and Ochteridae). The next branch represents one synapomorphy (19-3; SB2 absent) for Corixoidea and some species of Naucoridae and Helotrephidae. Inside of Corixoidea, one synapomorphy (2-4) for Micronectidae and one synapomorphy (36-2) for *Corixa dentipes* and *Stenocorixa protrusa* were distinguished. The absent SB2 was indicated in all presently studied Corixoidea (19-3).

On the next branch, characters (29-1) and (21-3) are not convincing because they are homoplasies. The indicated synapomorphic characters (9-3, 9-4 and 3-6) are convincing for Notonectidae, and only three species of Naucorinae are present among their members. The genera *Anisops* and *Notonecta* are indicated by one synapomorphy (9-4). They are the sister clade to *Buenoa* + *Enithares*, distinguished by synapomorphy (4-5). The species of notonectid *Martarega uruguayensis* possesses autapomorphy (7-3) and is connected with the species of Naucoridae (*Limnocoris volxemi* (19-1) and *Cryphocricos montei* (autapomorphies 2-3 and 3-9).

The Naucoridae are, at present, poorly diagnosed and synapomorphic characters are not visible for the family. In this reconstruction of characters, the synapomorphy (2-9) between the species *Limnocoris asper* and *Limnocoris pusillus* and two synapomorphies (6-1 and 8-2) for *Gestroiella limnocoroides* + *Ambrysus fuscus* are visible. The latter group is the sister to *Asthenocoris australis* by synapomorphy (23-6), and the clade of Pleidae is the sister by one synapomorphy (9-1) to a clade of *Helocoris strabus* + *Naucoris cimicoides* (with the common synapomorphy (23-7)). The clade *Plea minutissima* with *Paraplea sp*. possesses two synapomorphies (21-2 and 27-6). Moreover, Plea is distinguished by two autapomorphies (3-4 and 21-1).

The present cladistic analysis of the types and distributions of the antennal sensilla on particular antennomeres shows some sister relationships among taxa; nevertheless, most of the sensilla’s characters (autapomorphies) are spread across individual species of these families. Synapomorphic characters on the family level were indicated for Belostomatidae, Nepidae, Gelastocoridae, Ochteridae, Micronectidae, Pleidae and Notonectidae. Helotrephidae lacks significant relationships between *Hydrotrephes* and *Neotrephes*. The most varied characters are spread across individual species of Naucoridae. The family does not possess any obvious synapomorphy regarding the types and distributions of the sensilla’s characters. Individual characters of the sensilla in *Aphelocheirus aestivalis* (Aphelocheiridae) support its placement on a separate branch on the tree and indicate their advanced evolutionary level.

The Nepoidea’s position as a base taxon is rather confirmed by its sensilla’s characters, and the unique character is visible in some synapomorphies posted in the inner branches of the superfamily. It seems that Gelastocoridae and Ochteridae form one monophyletic group (Ochteroidea). One synapomorphy is present in all tested species of Corixoidea. The Pleidae species bring two synapomorhies.

The bootstrap analysis (Figure 8) shows that the character of the dataset is robust concerning the hypothesis of the monophyly of Nepomorpha (i.e., the clade is found in 100% of the trees). Nepoidea is indicated in 40% (low support), while Nepidae is found in 74% but most species of Belostomatidae in 46%. The high rate of support is maintained for the subfamily Lethocerinae: 91%. The attainment of the above 70% bootstrap level is observed in only a few clades: Micronectidae (82%), *Sigara nigrolineata* + *Sigara striata* (74%) (Corixidae), *Helocoris strabus* + *Naucoris cimicoides* (70%) (Naucoridae), *Plea minutissima* + *Paraplea* sp. (Pleidae) (78%) and *Enithares metallica* + *Enithares stridulata* (75%) (Notonectidae).

There was no bootstrap support (attainment of below 70% bootstrap level) noticed for the hypothesized relationships between the representatives of the remaining families/species. In contrast to the consistent placement of these taxa in the shortest tree, this lack of bootstrap support reflects the fact that relatively few (although highly consistent) characters support the nodes.

## 4. Discussion

The present study characterizes the antennal sensillar equipment across Notonectoidea and summarizes the types and distribution of sensilla on representatives of other nepomorphan superfamilies. It is the first large comparative study of the antennal sensilla of Nepomorpha to provide data on the diversity of antennal morphology and sensillar equipment (Figures S1–S4).

However, some of the previous studies on nepomorphan antennal sensilla deal with only one or a few species [32], making it difficult to compare sensilla within taxonomic groups. Ultramorphology on the antennal sensilla of the nepomorphan superfamily has been conducted systematically in several studies [24,25,26,27], using a consistent terminology for the sensilla types. A previous study on the antennal sensilla of one family of Pleidae (Notonectoidea) was presented by Garza et al. [33]. The basic problem when comparing the sensillar types of some taxa of Nepomorpha is the different classification of the sensilla used by the mentioned authors.

### 4.1. Morphology and Categories of the Antennal Sensilla of Notonectoidea

Most of the studied species of Notonectidae show a similar set of antennal sensilla.

Sensilla trichoidea (ST) are classified as mechanosensilla and are the most diverse sensillar structures, ranging from thin hair-like to specific leaflike sensilla with a ribbed surface. In the superfamily Notonectoidea, five morphological types (ST1, ST2, ST3, ST4 and ST5) that occur in almost all species of the family Notonectidae were recognized (Table 1), with different distributions and density on particular antennomeres. The most significant feature observed in Notonectidae are the long, flattened sensilla trichodea (ST4) on the third and fourth antennomere, which protrude above all other sensilla (Figure 4c,f). ST4 are only absent in the *Enithares* species. Chaika and Sinitsina [32] found a few types of sensilla trichodea in *Notonecta glauca*, including the oar-like sensilla that is designated to ST4 in our studies. Such extended (in comparison to the rest of sensory structures) sensilla have not been observed in previously studied nepomorphan taxa [24,25,26,27]. Their shape and size suggest they might be used to recognize the vibrations of water waves.

This current study of Pleidae pointed to the three types of sensilla trichoidea (ST1, ST2 and ST5). These types of sensilla are compared with the trichoid and leaf-like sensilla of the pleid species distinguished by Garza et al. [33]. The results are shown in Table 3.

In the two studied species of Helotrephidae, the set of sensilla are quite different: *Hydrotrephes visayasensis* possesses ST1, ST2, ST3 and ST5, in contrast to *Neotrephes lanemeloi* which has only ST1.

The second characteristic type of mechanosensilla is the leaf-like sensillum ST5, which is present in all studied species except in two species of *Enithares* (Notonectinae) and *Neotrephes lanemeloi* (Helotrephidae). This sensillum has a very wide surface but is significantly shorter than ST4. The sensilla ST5 in Notonectoidea are similar to: SPL1 in Nepoidea [24], SCIL in Ochteridae and Gelastocoridae [25], ST3 in some species of Naucoridae [27] and Sl2 in Pleidae [33]. Some differences are present in the shapes and sizes of mechanosensilla, making it difficult to find an exact match between multiple taxa.

The leaf-like sensilla trichodea type ST3 is a rare type of sensillum in the nepomorphan taxa. It was distinguished in *Lethocerus indicus* (Belostomatidae) [24] and in a few species of Corixidae [26]. In Notonectoidea, type ST3 is present only in three genera (*Anisops, Buenoa* and *Enithares*) as well as in *Hydrotrephes* (Helotrephidae). The ST1 and ST2 distinguished in most species of Nepomorpha belong to a conservative group of mechanosensilla.

Sensilla chaetica (SCh), sensilla basiconica (SB1) and sensilla campaniformia (SCa) are part of the mechanosensillar group in Notonectoidea, similar to other nepomorphan species [24,25,26,27]. SCh is a sensillum found only on the second antennal segment of the three notonectid species investigated. This sensillum is a stout hair that does not have pores and is not numerous. Regarding its morphology, it functions as a mechanoreceptor [38].

Generally, sensilla campaniformia are commonly present in insects and are situated in areas of the cuticle that are subject to stress [20]. These sensilla are found on different parts of the antennae, and have usually been reported on the scape, near the segmental joints or the membrane on the top of the pedicel [39]. In some species of Notonectidae and Helopthephidae, one type of sensillum campaniformia was photographed in SEM; we assume that it is present in all species. Similarly, the sensilla basiconica (SB1) probably occurred in most studied species; and were documented in *Notonecta, Enithares, Martarega, Paraplea, Hydrotrephes* and *Neotrephes*. In some of the studied species, sensilla basiconica may be hidden given their usual place of occurrence (between the antennomeres).

The system of chemosensilla on the antennae of the Notonectoidea is represented by four types of olfactory sensilla (SB2, SB3, SB4 and SB5). According to the external morphology, after Slifer and Sekhom [40] and Steinbrecht and Müller [41], SB2 and SB3 represent thin wall porous olfactory sensilla of different lengths. SB2 is shorter and SB3 is longer. The long sensillum basiconicum is characteristic for most of the studied Notonectidae species, except in Hydrothephidae and in the *Martarega* species.

The morphology and ultrastructure of some types of the sensilla in *Notonecta glauca*, presented by Chaika and Sinitsina [32], showed that structures described as sensilla trichodea with an abrupt ending are in fact built like the olfactory sensilla. This sensillum corresponds to the long sensilla basiconica (SB3), which is also found in *N. glauca* and other notonectid species. This sensillum is the dominant type in the Naucoridae, present singularly in *Ochterus, Gelastocoris* and *Aphelocheirus* and numerous in Nepoidea, distributed on the second and third antennomeres.

SB4 has a partly ribbed surface with pores between the ribs, and is present in some notonectid species (Table 2). This olfactory sensillum is classified as a thick-walled sensillum (Figure 4d and Figure 5a). In the present classification, SB4 is present in Corixidae (SB with the partly ribbed surface and porous), in Ocheteridae, Gelastocoridae, Aphelocheiridae and Naucoridae [25,27], but not in Nepoidea. The only chemosensillum type found in *Martarega uruguayensis* (Figure 4b) and *M. gonostyla* was the SB5 (thin-walled). No other types of chemosensilla basiconica were found in these species. SB5 is also present in a few other notonectid species and Plea, along with SB2, SB3 or SB4. A common type of chemosensillum is SB2, recognized in *Anisops, Notonecta, Enithares* and *Paraplea*. It was found across most other nepomorphan species in Nepoidea, Ocheteridae, Gelastocoridae, Aphelocheiridae and Naucoridae, except for the Corixidae (Table 4).

The thermo-hygroreceptive sensilla of the studied species were classified into two subtypes: sensilla coeloconica SCo1 and SCo2, with a slight difference in shape. These sensilla are present in most studied species (Table 2) and are usually located on the two last antennomeres. Sensilla coeloconica SCo were absent only in Helotrephidae. However, the same function is probably performed by sensilla ampullacea. Whatever the case, the thermo-hygroreceptive ability in insects plays an essential role in the selection of habitats and is widely documented. When compared with the external appearance of the functionally identified sensilla of other insects, hygro-thermosensitive sensilla are recognized as small, aporous cuticular pegs embedded in shallow concaves (i.e., sensilla coeloconica) or set in pits (sensilla ampullacea) [42]. Moreover, Altner et al. [43] suggested that such morphological characters of the sensillum (aporous and seated in an inflexible socket) represent a sensillum whose structural features can be regarded as adaptations to thermo-hygoreception. From previous studies, it follows that the nepomorphan species possess thermo-hygrosensilla on their antennae, and that they can be used to assess water temperature and humidity in habitats [24,25,26,27,32,33].

### 4.2. Comparison of the Morphological Characters and Distributions of Antennal Sensilla in the Nepomorphan Taxa

The aim of the cladistic analysis was to find an answer to the research hypotheses by examining and estimating the morphological features of antennal sensilla. Therefore, the authors used the MP tree to recognize the sensilla characters. The sensilla were tested to indicate the apomorphic characters in particular taxa, whereas the comparison of the relationships between the clades (Figure 7) is less important and therefore secondary in this analysis due to the small group of morphological features.

The monophyly of Nepomorpha is widely accepted [6,8,44,45,46].

The last comprehensive study conducted by Ye et al. [8] includes many taxon samples (115) for molecular and morphological reconstruction, divergence time estimation and diversification analyses of the infraorder Nepomorpha. Their study provides a robust reconstruction of phylogenetic relations and revises the previous phylogenetic hypothesis proposed by the authors mentioned above.

The present cladistic analysis of the antennal sensilla of the majority of nepomorphan families (except Potamocoridae) includes 57 species, and hypothesizes their relationships based on estimated synapomorphies (Figure 7). These hypotheses are compared to the phylogenetic tree proposed by Ye et al. [8].

Based on the analysis of its antennal sensilla, the position of Nepoidea on the MP tree (Figure 7) is not questionable regarding its previous placement [8]. The characteristic feature of Nepoidea is the presence of SB3c (no. 22) (sensillum basiconicum that rises from inflexible sockets, is bent towards the antenna, and has a porous surface). In Belostomatidae, the sensillum is present only on the third and fourth antennomeres (except in *Lethocerus*), whereas in Nepidae, SB3c is present on the second and third antennomeres. The second common characteristic for these families is the presence of sensilla coeloconica SCo3, with the same arrangements as the ones noted for SB3c.

Belostomatidae probably has a synapomorphy with Nepidae, as sensilla coeloconica SCo2a are present only on the fourth antennomere in *Lehocerus*, and present on the third antennomere in *Hydrocyrius* (no. 25-1). These sensilla were also observed in some species of Nepidae (no 25-2). Nepoidea (Table 4) is evaluated as a monophyletic superfamily (Figure 7), given that the aforementioned types of sensilla are present only across this group. This status of superfamily was previously recognized by Ye et al. (2019) and other, earlier authors. In addition to the stated synapomorphies, some species have characteristic features (autapomorphies), significantly different types of mechanosensilla (sensilla cone-like SCoL (11-1), sensilla brush-like SBL (12-1), and/or sensilla paddle-like SPL1 (14-1, 14-2) (Figure 7, Table 4). Within Nepoidea, the families Belostomatidae and Nepidae were recognized as monophyletic groups [8].

In this analysis, Aphelocheiridae is represented by one species with one autapomorphy (sensilla plate-like SPl). This character was only found in this taxon. Its position on the present tree is very weakly supported and drawn close to the branch of Nepoidea, probably due to plesiomorphic/homoplastic characters such as the absence of sensilla trichoidea ST1 (which are also absent in *Lethocerus indicus*, *Lethocerus patruelis* and in nepid species). Thus, this sensillum (SPl) does not matter in relationships between family taxa. No synapomorphy was found in the present analysis on the basis of the other sensilla types of Naucoridae and Aphelocheiridae.

In the last phylogenetic analysis, Aphelocheiridae was placed as a sister group to Naucoridae together with Potamocoridae, and these taxa formed the superfamily Naucoroidea as the most advanced taxon in Nepomorpha [8].

Ochteroidea (Ochteridae and Gelastocoridae) is treated as a monophyletic group and is supported by two synapomorphies: sensilla SB2, present only on the antennomeres 2-4, and especially the presence of sensilla placodea, multilobated SPM. The position of the Ochteroidea is not in congruence with Ye et al., [8], Brożek [6] and Hebsgaard et al. [4]. However, it is more important to find synapomorphic characters in the present analysis. Similarly, the Helotrephidae occupy other positions on the MP tree (Figure 7) than the positions suggested by Ye et al., [8], Brożek [6] and Hebsgaard [4]. Nevertheless, the autapomorphic sensilla characters in two species of different genera (*Hydrotrephes visayasensis* 4-4, 9-2 and *Neotrephes lanemeloi* 1-3, 19-6) and their synapomorphy (1-2) were noted.

The next node included Corixoidea as a sister group to some Naucoridae (with an inner branch composed of Pleidae and Notonectidae). The Corixoidea have putative synapomorphy (19-3, the sensilla basiconica SB2 absent) and form a monophyletic group. The family Micronectidae reveals autapomorphy (sensilla trichoidea ST1, present only on the third antennomere), which further strengthens the status of this family. It reinforces the findings of Nieser [47] treating the studied taxa as two separate families: Micronectidae and Corixidae.

In addition, one synapomorphy between *Corixa dentipes* and *Stenocorixa protrusa* has been demonstrated. In all Corixoidea, there are three sensillar apomorphies which may indicate a very homogeneous specialized group, especially when the diversity of its sensilla types was the smallest, when compared to other nepomorphans [26].

The types and distributions of antennal sensilla are strongly differentiated among naucorid species and no sensillar synapomorphies were found for Naucoridae. Only a few synapomorphies were indicated for some subfamilies. The two species of the subfamily Limocorinae possesses one synapomorphy (7-9), whereas another species (*Limnocoris volxemi* autapomorphy (19-7)) is sent to the family Notonectidae. *Gestroiella limnocorides* (Cheirochelinae) shares a common synapomorphy (6-1, 8-2) with *Ambrysus* *fuscus* (Cryphocricinae). However, they represent different subfamilies. A similar case is observed between *Helocoris strabus* (Laccocorinae) and *Naucoris cimicoides* (Naucorinae). These species share a synapomorphy (23-7), while *Naucoris scutellaris* was placed on the branch common with the Notonectidae. In this study, Pleidae is distinguished by two synapomorphies (21-2; 27-6), but they are placed in the inner taxon in Naucoridae, due to one synapomorphy (9-1) with the clade *Helocoris strabus* and *Naucoris cimicoides*. Such position of the Pleidae is not supported by other studies, which seems obvious.

In some papers, Pleidae is recognized as a sister group to Helotrephidae and together they are placed as a sister group to Notonectidae [3,6]. The last phylogenetic analysis by Ye et al. [8] strongly documented Pleoidea (Pleidae + Helotrephidae) as the sister taxon to Notonectoidea.

The Notonectidae clade appears with one synapomorphy (9-3, ST5 (b) only on the third antennomere). One branch with synapomorphy (9-4) includes the *Anisops* and *Notonecta* species. In another branch, represented by some notonectids, species share synapomorphic characters (ST2 present only on the first and second antennomeres) with three species of Naucoridae. *Enithares* species and *Buenoa* are grouped via synapomorphy on the inner branch (4-5). The *Enithares* clade is evidently distinguished by two synapomorphies (4-6, 20-4). Moreover, one species possesses one autapomorphy (28-2) whereas the second has three autapomorphies (2-7, 3-7, 20-6). Furthermore, *Martarega* possesses autamomorphy (7-3) and is placed between naucorid species (*Limnocoris volxemi* and *Cryphocricos montei*). Such a distribution of features questions this taxon as monophyletic. Nevertheless, previous studies recognized it as monophyletic on the basis of extensive phylogenetic analyses [8].

The current cladistic analysis (Figure 7) showed that the types and distributions of the antennal sensilla in Nepomorpha are highly varied. Most of the sensilla are plesiomorphic/homoplastic characters of no phylogenetic importance for the indication of closely related monophyletic taxa. Nevertheless, based on the present analysis, we have documented the sensilla’s apomorphic characters in some nepomorphan taxa, which can be used in additional future analyses. Presently, some synapomorphies have been diagnosed, and the monophyletic superfamilies of Nepoidea, Corixoidea and Ochteroidea have been supported. Presumably, we can also accept synapomorphies for all Notonectidae, excluding the three species of naucorids. The monophyletic families clearly identified based on antennal sensilla are Nepidae, Aphelocheiridea, Micronectidae, Gelastocoridae, Ochteridae and Pleidae. In lower taxa, the sensillar autapomorphic characters are numerous (22) in particular species.

Previously, there were studies on the morphology of labial sensilla which provided data to discuss the phylogenetic links within all nepomorphan taxa, especially at the level of family/subfamily or tribe [48]. The analysis of the characters of the types of these sensilla (and their distribution on the labium) has provided specific valuable systematic information regarding the subfamilies or families and allowed insights into the complexity of character evolution, e.g., cupola and peg sensilla were recognized as plesiomorphic features of the Belostomatidae; the paddle-like sensillum in the Ranatrinae, the squamiform sensillum in the Nepinae, the club-like sensillum in the Gelastocorinae, the chaetica sensillum with a bisected tip in the Nerthrinae, the star-like sensillum in the Aphelocheiridae, and finally the multilobe sensillum in some of the Naucoridae were autapomorphies for each one of these, whereas the ribbon-like sensillum was a synapomorphy for the Corixidae, Micronectidae, and Diaprepocoridae [48].

As shown by the above data, some features (shape, size and distribution) of antennal sensilla in a certain range of taxa show phylogenetic value as synapomorphies and apomorphies similar to some of the analyzed sensilla on the labium.

### 4.3. Types of Sensilla in Nepomorphan Families

The antennal sensilla of insects play a key role in perceiving external information [49,50]. Touch, physical pressure, movement, stretching, vibrations, and contractions all serve to alter the position of the cuticle of the mechanosensilla [38], whereas chemosensilla are responsible for the recognition of environmental chemistry [19]. Thermo-hygrosensilla control water balance and sense humidity and temperature variations [51].

Our previous [24,25,26,27] and current analysis of particular families were done to assess whether, given that the nepomorphan species are a secondary group adapted to water life, their olfactory sensors and the shape of their mechanosensilla are morphologically changed for water habitats [2].

The diversification of nepomorphan superfamilies is widely associated with the evolution of biological and behavioral innovations such as air-breathing through siphons in Nepoidea, back-swimming in Pleoidea and Notonectoidea, and plastron respiration in Aphelocheiridae and some members of Naucoridae [8]. Therefore, the antennal sensillar system is also regarded as an important element of adaptation to the aquatic environment of the studied group.

The development of the superfamilies and families of Nepomorpha occurred between the Late Permian and Jurassic, after the end-Permian mass extinction caused lentic habitats to change to new habitats (i.e., lotic and riparian), increased the availability of new habitats, and led to continuous radiation of nepomorphan species with heterogeneous diversification rates. The high diversification rate occurred in the early stages of the establishment of superfamilies and families until the Late Jurassic, around 159 Ma. However, most extant genera evolved at a decreased rate.

According to Ye et al. [8], at least six independent transitions from lentic to other habitats occurred during the evolution of some nepomorphans, including one transition to riparian habitats which occurred at the onset of Ochteroidea. Moreover, in gelastocorids and ochterids (Ochteroidea), the terrestrial way of life is assumed to have evolved secondarily [4]; as such, they possess unique morphological characters, e.g., the thoracic air stores not found in other terrestrial bugs and shortened antennae.

Thus, we must ask whether the complexity and importance of mechanical and chemical stimuli relevant to water/riparian species leads to the differentiation of sensilla types, and to their number. Universally, a similar sensillar system is kept throughout the different insect orders, making it improbable that these different types of sensilla evolved independently several times [52]. That is probably why a similar set of functional sensilla is observed in terrestrial and aquatic heteropteran insects. However, distinct particular characteristics of sensilla were observed in different taxa.

The different forms of mechanosensilla trichoidea (ST1-ST5), leaf-like, and chaetica (SCh) in nepomorphan families are compared below. The sensilla basiconica SB1 and campaniformia each have a single shape. The ampulacea and aporous coeloconica sensilla, treated as thermo-hygroreceptors, were present in most studied nepomorphan taxa.

Within Nepomorpha, the Nepoidea possessed the greatest diversity of mechanosensilla in general. Eleven types of mechanosensilla were found in this superfamily, whereas six types were found in Ochteroidea. Aphelocheiridae is very poor regarding such sensilla; three basal types were found (ST2, SCa and SB1) [25]. The families Corixidae and Micronectidae have strong uniformity in patterns of sensilla trichodea (ST1-ST4), and one campaniform sensilla [26]. Naucoridae presents six types of mechanosensilla [27], similar to Notonectidae. Pleidae and Helotrephidae possess four types of mechanosensilla each.

In comparison to nepomorphan bugs, five types of antennal mechanosensilla have been found in Gerromorpha and six types were pointed to in Heteroptera [35]. Antennal olfactory chemosensila (basiconica, placodea) were also confirmed in Gerromorpha and other heteropterans [35]. The single-walled wall-pore olfactory sensilla basiconica (SB2-SB3) and sensilla coeloconica (SCo1-SCo3) were documented in Nepoidea [24].

Olfactory sensilla placodea multilobated (SPM; single-walled wall-pore) were reported only in the species of Gelastocorinae and Nerthrinae. Moreover, two groups of olfactory sensilla basiconica SB2-SB3 (single-walled wall-pore) and sensilla basiconica SB4 (double-walled wall-pore) were reported in Ochteroidea [25]. Aphelocheiridae possesses the olfactory plate-like (single-walled wall-pore) sensilla. In Corixoidea, only one type of olfactory sensillum (double-walled wall-pore) was indicated as SB (equivalent to SB4 in other taxa). Three types of olfactory sensilla basiconica SB2-SB3 (single-walled wall-pore) and SB4 (double-walled wall-pore) were documented in some species of Naucoridae. In Notonectidae and Pleidae, there was one more type of sensillum (SB5, single-walled wall-pore) than in Naucoridae. Only one sensillum basiconium SB2 (single-walled wall-pore) was confirmed in Helotrephidae. In some taxa there are olfactory sensilla (single-walled wall-pore, double-walled wall-pore, or both). Steinbrecht [52] claimed that these two types of olfactory sensilla evolved independently. The inspection of nepomorphan sensilla reveals a diverse system of olfaction. All sensilla categorized to the olfactory system meet the standards regarding olfactory sensilla morphologically documented in other insects by many authors previously [21,24,25,26,27,34].

The morphological differences, especially the evidently flattened types of mechanosensilla (ST3, ST5, ST5, SBL, SCIL, SPL1, SPL2, SSq) and significant variation in the number of mechanosensilla (some are localized on special places on the antennomeres) in the nepomorphans relate to their aquatic lifestyle. It is also known that typical sensilla trichodea ST1, ST2, campaniformia SCa and basiconica proprioceptive SB1, which are present in nepomorphan taxa, are also widespread in terrestrial insects, e.g., heteropterans [35,53,54,55,56].

The reduced number of sensilla in general when compared to terrestrial insects may be an extreme adaptation for the aquatic environment. The antennae of *Aphelocheirus* are situated on the head, without any physical protection from environmental stressors, and therefore not concealed in grooves beneath the eyes as observed in Gelastocoridae or Nepoidea [2,24,25]. Thus, we suppose that the absence of long mechanoreceptive sensilla on the antennal surface of *Aphelocheirus* (a rheophile, that is, an inhabitant of fast-flowing small rivers) might be an adaptation to the type of environment.

The olfactory and thermo-hygroreceptive antennal systems show, as a rule, a smaller range of morphological diversity among the different nepomorphan taxa, and those sensilla are frequently very similar to other heteropterans [57,58,59]. The most important factor is that nepomorphans are secondary aquatic organisms, and some modifications of their mechanosensilla and to a lesser extent, their chemosensilla (as seen in Aphelocheiridae or Gelastocoridae) can be a response to the aquatic environment.

## 5. Conclusions

A significant degree of variability in the morphology of antennal sensilla (shape, length, number and position on the antennomeres) was observed within all Nepomorpha. Antennal sensilla are represented by five types of sensilla trichodea, two types of sensilla leaf-like, one type of sensilla paddle-like, cone-like, squamiformia, brush-like and club-like, one type of sensilla chaetica, one type of sensilla campaniformia, five types of sensilla basiconica with different functions, four types of sensilla coeloconica with different functions, sensilla ampullacea, one type of sensilla placodea multilobate, and sensilla plate-like.

Such variability makes it difficult to propose the general use of sensillar characters in taxonomic studies. However, at least some characteristics may help distinguish some families and genera with special features on the antennae (e.g., *Nepa* with sensilla squamiformia, Notonecta with very long sensilla trichodea ST4, and others mentioned in this paper). 

Some evolutionary trends for certain sensilla types are suggested, such as sensilla squamiformia in Nepidae, plate-like sensillum present only in *Aphelocheirus*, and sensilla placodea multilobated in Gelastocoridae. Such sensilla are absent in other taxa.

Our study of antennal sensilla using a cladistic analysis evidences synapomorphies for several families (Belostomatidae, Nepidae, Micronectidae, Corixidae, Gelastocoridae, Ochteridae, Notonectidae and Pleidae).

These results suggest that the antennal sensilla of Nepomorpha evolved in inner groups. The Nepidae and Belostomatidae (Nepoidea) were characterized by numerous types of mechanosensilla (nine main types and three subtypes). In contrast, the number of mechanosensilla types in other groups was significantly lower: three-four in Ochteridae and Gelastocoridae, two in *Aphelocheirus,* five types in Corixoidea, six types in Naucoridae and Notonectidae, four types in Pleidae, five types in *Hydrotrephes* and two in *Neotrephes*. The olfactory chemosensilla (sensilla basiconica SB2, SB3 and SB4) were more common in most nepomorphan taxa. The olfactory sensilla coeloconica (SCo1, SC2 and SCo3) were specific for Nepidae, while sensilla placodea SPM were noted in Gelastocoridae and SPL in Aphelocheiridae.

In addition, essential attributes in our analysis are the numerous autapomorphies reported in particular species, indicating the evolutionary changes in the sensilla.

On the other hand, the observed variability, especially within mechanosensilla, may be linked to different life-history traits, most notably the several (at least six) independent transitions from lentic to other habitats that occurred during the evolution of Nepomorpha. This high variability in mechanosensilla indicates their greater adaptability to the aquatic environment, as it is not entirely the same for individual taxa. The species representatives of nepomorphan taxa occur in rushing streams (e.g., Aphelocheiridae), free-flowing waters (e.g., Nepidae, Belostomatidae, Corixidae, Notonectidae, Naucoridae, Pleidae and Helotrephidae) and in almost terrestrial environments (Ochteridae and Gelastocoridae).

The chemosensory antennal sensilla (their shapes and distributions) could be more meaningful in the phylogenetic analysis of particular taxa, because they represents less variability than mechanosensilla.

The thermo-hygroreceptive sensilla represent two unchanging morphological types (coeloconic and ampullacea) in most of the studied nepomorphan taxa. The proprioceptive sensillum basiconium (SB1) and mechanoreceptive sensillum campaniformium are almost identical in all studied taxa, and probably represent plesiomorphic characters, similar to the case of thermo-hygoreceptive sensilla.

Nevertheless, a morphological and functional adaptation of some sensilla is commonly observed in nepomorphans as well as a characteristic convergence, and may prevent an objective understanding of their systematics and evolution.

## Figures and Tables

**Table 1:** Matrix characters (57 taxa and 32 characters multistate).

Gerromorpha	00000000000000000000000000000000
*Belostoma*_*elegans*	01120000010000100010011001100001
*Diplonychus*_*annulatus*	01220000030000000110011001101001
*Hydrocyrius*_*colombiae*	01220000020000000110011111100001
*Lethocerus*_*indicus*	02210000041000000010001010100001
*Lethocerus*_*patruelis*	02210000040000100110001010100001
*Nepa*_*cinerea*	11220000010100110020021002100001
*Laccotrephes*_*fuscus*	12220000010000010120021002101001
*Laccotrephes*_*fabricii*	12220000010001010120021022100001
*Ranatra*_*grandocula*	12120000010010000120021002100001
*Ranatra*_*linearis*	12120000010002000120001022101001
*Ranatra*_*unicolor*	12120000010001000120001022100001
*Agraptocorixa*_*hirtifrons*	03030000010000001130002000201001
*Callicorixa*_*praeusta*	03021000010000001130003000201001
*Corixa*_*affinis*	03021000010000001130003000201001
*Corixa*_*dentipes*	03021000010000001130004000300001
*Corixa*_*punctata*	03021000010000001130002000201001
*Paracorixa*_*concinna*	03021000010000000130002000201001
*Sigara*_*nigrolineata*	03031000010000000130002000200001
*Sigara*_*striata*	03031000010000000130002000200001
*Cymatia*_*coleoptrata*	03021000010000001130002000201001
*Stenocorixa*_*protrusa*	03021000010000001130004000200001
*Micronecta*_*poweri*	14130000010000001130002000101001
*Micronecta*_*scholtzi*	14131000010000001130002000101001
*Aphelocheirus*_*aestivalis*_	02320000010000000110001000100101
*Ochterus*_*marginatus*	01020000000000001001005000300001
*Gelastocoris*_sp.	01020000010020001042003000100011
*Gelastocoris*_*flavus*_*flavus*	01020000010020000040003000400011
*Nerthra*_*femoralis*	01020000010010000040001000500011
*Nerthra*_*fuscipes*	01020000010000001040001000100011
*Nerthra*_*ampliata*	01020000010000001040001000100011
*Nerthra*_*ranina*	01020000010000001040005000500011
*Plea*_*minutissima*	11420000110000001133106000601001
*Paraplea*_sp.	11120000110000000010206000601001
*Hydrotrephes*_*visayasensis*	21240000210000000130001000100001
*Neotrephes*_*lanemeloi*	31120000010000000150001000101001
*Anisops*_*jaczewski*	11020010410000000160302000111001
*Buenoa*_*nitida*	13650010310000000133301000101001
*Enithares*_*metallica*	06060000010000001134400000121001
*Enithares*_*stridulata*	07760000010000000035302000111001
*Notonecta*_*ceres*_*ceres*	01220020540000000012302000510001
*Notonecta*_*disturbata*	01020020510000000032301000111001
*Notonecta*_*glauca*	01020020740000001110401000511001
*Notonecta*_*maculata*	01020010540000001110501000111001
*Martarega*_*uruguayensis*	05830030710000000133001000510001
*Asthenocoris*_*australis*	02020001010000000130406000501001
*Gestroiella*_*limnocoroides*	02120102010000000120501000500001
*Ambrysus*_*fuscus*	02820202010000000100401000501001
*Cryphocricos*_*montei*	08920000010000000100000000500001
*Heleocoris*_*strabus*	05220000610000000130407000501001
*Limnocoris*_*asper*	09020000010000000130401000101001
*Limnocoris*_*pusillus*	19320000010000000130302000201001
*Limnocoris*_*volxemi*	01620000610000000170402000501001
*Naucoris*_*cimicoides*	06020000510000001110507000301001
*Naucoris*_*scutellaris*_	01620000510000000100301000301001
*Pelocoris*_*femoratus*	05220000610000000060400000201001
*Pelocoris*_*poeyi*	05220000010000000060400000301001

**Table 3:** The different types of sensilla recognized in *Plea minutissima*.

Sensilla Types	Present Study *Plea minutissima*	Study of Garza et al. (2021) *Plea minutissima*
Mechanoreceptive sensilla	ST1—hairlike sensillum with a smooth surface and a flexible socket ST2—hairlike sensillum with a ribbed surface and a flexible socket ST5—leaflike sensillum with a flexible surface	ST1—long (40–70 µm) hairlike structures with an inflexible socket ST3—sensilla with the length of 40–55 µm, curled towards the apex with an inflexible socket SL2—leaflike sensilla, wide and long with a flexible socket SL3—the widest leaflike sensilla with a ribbed surface and a flexible socket SCh—short, thick and hairlike sensilla with a flexible socket
Chemoreceptive sensilla	SB3—long porous structures with an inflexible socket SB4—short cones with a smooth surface on the base and the rest of the sensillum ribbed SB5—short flattened cones with an inflexible surface	SUT—hairlike sensilla with a single pore on the surface with an inflexible socket SB—cones with an apical pore, smooth at the base with channels extending to the apical pore
Thermo-hygroreceptive sensilla	SCo1—pegs arising from the cuticle, sometimes on a protuberance of the cuticle with an inflexible socket	SCo1—pegs arising from a pit in the center of a cone with a movable socket SCo3—pegs arising from a simple pit

**Table 4:** The presence of all types of sensilla and their functions recognized in Nepomorpha, based on the papers of Nowińska and Brożek [24,25,27] and Nowińska et al. [26]. The additional letters (a,b,c) associated with the types of sensilla, (e.g., ST4a) correspond to different morphological types named the same in different papers due to numeration.

Family/Species		Mechanoreception																	Chemoreception								Thermo-Hygroreception			Unknown Function	
		ST1	ST2	ST3	ST4a	ST4b	ST4c	ST5a	ST5b	SCh	SCoL	SBL	SClL	SPL1	SPL2	SSq	SCa	SB1	SB2	SB3a	SB3b	SB3c	SB4	SCo1a	SCo2a	SCo3	SCo1b	SCo2b	SA	SPl	SPM
Belostomatidae	*Belostoma elegans*	+													+		+	+	+			+				+			+		
	*Diplonychus annulatus*	+	+							+							+		+			+				+					
	*Hydrocyrius colombiae*	+	+							+							+		+			+		+	+	+			+		
	*Lethocerus indicus*		+	+						+	+						+	+	+						+				+		
	*Lethocerus patruelis*		+	+						+					+		+		+						+				+		
Nepidae	*Nepa cinerea*	+	+									+			+	+	+	+	+			+				+			+		
	*Laccotrephes fuscus*		+													+	+		+			+				+					
	*Laccotrephes fabricii*		+											+		+	+		+			+			+	+			+		
	*Ranatra grandocula*												+				+		+			+				+			+		
	*Ranatra linearis*													+			+		+						+	+					
	*Ranatra unicolor*													+			+		+						+	+			+		
Aphelo cheiridae	*Aphelocheirus aestivalis*		+														+		+	+									+	+	
Ochteridae	*Ochterus marginatus*	+	+							+								+	+	+			+				+		+		
Gelastocoridae	*Gelastocoris* sp.	+	+										+					+	+	+			+						+		+
	*Gelastocoris flavus flavus*	+	+										+				+	+	+				+				+		+		+
	*Nerthra femoralis*	+	+										+				+	+	+								+		+		+
	*Nerthra fuscipes*	+	+															+	+										+		+
	*Nerthra ampliata*	+	+							+								+	+										+		+
	*Nerthra ranina*	+	+															+	+				+				+		+		+
Corixidae	*Agraptocorixa hirtifrons*	+	+	+																			+				+				
	*Callicorixa praeusta*	+	+		+																		+				+				
	*Corixa affinis*	+	+		+																		+				+				
	*Corixa dentipes*	+	+		+																		+				+		+		
	*Corixa punctata*	+	+		+																		+				+				
	*Paracorixa concinna*	+	+		+												+						+				+				
	*Sigara nigrolineata*	+	+	+	+												+						+				+		+		
	*Sigara striata*	+	+	+	+												+						+				+		+		
	*Cymatia coleoptrata*	+	+		+																		+				+				
	*Stenocorixa protrusa*	+	+		+																		+				+		+		
Micronecidae	*Micronecta poweri*	+		+																			+								
	*Micronecta scholtzi*	+		+	+																		+								
Naucoridae	*Asthenocoris australis*		+					+									+				+		+				+				
	*Gestroiella limnocoroides*					+		+									+		+		+						+		+		
	*Ambrysus fuscus*		+			+		+									+		+		+						+				
	*Cataractocoris marginiventris*	+	+			+		+									+				+		+								
	*Heleocoris strabus*	+	+						+								+				+		+				+				
	*Limnocoris asper*	+	+														+				+										
	*Limnocoris pusillus*	+	+														+				+		+				+				
	*Limnocoris volxemi*	+	+						+								+		+		+		+				+				
	*Naucoris cimicoides*	+	+						+										+		+		+				+		+		
	*Naucoris scutellaris*	+	+						+								+		+		+						+				
	*Pelocoris femoratus*	+	+						+								+	+	+		+		+				+				
	*Pelocoris poeyi*	+	+														+	+	+		+		+				+		+		
Notonectidae	*Anisops jaczewski*	+	+				+		+								+		+		+		+					+			
	*Buenoa nitida*	+	+	+			+		+								+			+	+										
	*Enithares metallica*	+	+	+																+	+		+					+			
	*Enithares stridulata*	+	+	+													+	+	+	+	+		+					+			
	*Notonecta ceres ceres*	+	+				+		+	+							+	+	+	+	+		+				+	+	+		
	*Notonecta disturbata*	+	+				+		+								+	+		+	+							+			
	*Notonecta glauca*	+	+				+		+	+									+		+						+	+			
	*Notonecta maculata*	+	+				+		+	+									+		+							+			
	*Martarega uruguayensis*	+	+	+			+		+								+			+							+	+	+		
Pleidae	*Plea minutissima*	+	+						+											+	+		+				+				
	*Paraplea* sp.	+							+								+	+	+		+		+				+				
Helotrephidae	*Hydrotrephes visayasensis*	+	+	+					+								+												+		
	*Neotrephes lanemeloi*	+															+		+												

## Data Availability

Not applicable.

## Supplementary Materials

The following are available online at https://www.mdpi.com/article/10.3390/insects12121121/s1, Figure S1: Schematics of the types of sensilla in Nepoidea, Figure S2: Schematics of the types of sensilla in Ochteridae, Gelastocoridae and Aphelocheiridae, Figure S3: Schematics of the types of sensilla in Corixidae and Micronectidae, Figure S4: Schematics of the types of sensilla in Naucoridae.
