# Supplementary material for: Morphology of the Antennal Sensilla of Notonectoidea and Comparison of Evolutionary Changes in Sensilla Types and Distribution in Infraorder Nepomorpha (Insecta: Heteroptera)
Source: Insects. 2021 Dec 14;12(12):1121. doi: 10.3390/insects12121121 (PMC8703933; doi:10.3390/insects12121121)
Supplement: Supplementary file 1 [file insects-12-01121-s001.zip › insects-1499818-supplementary.pdf]

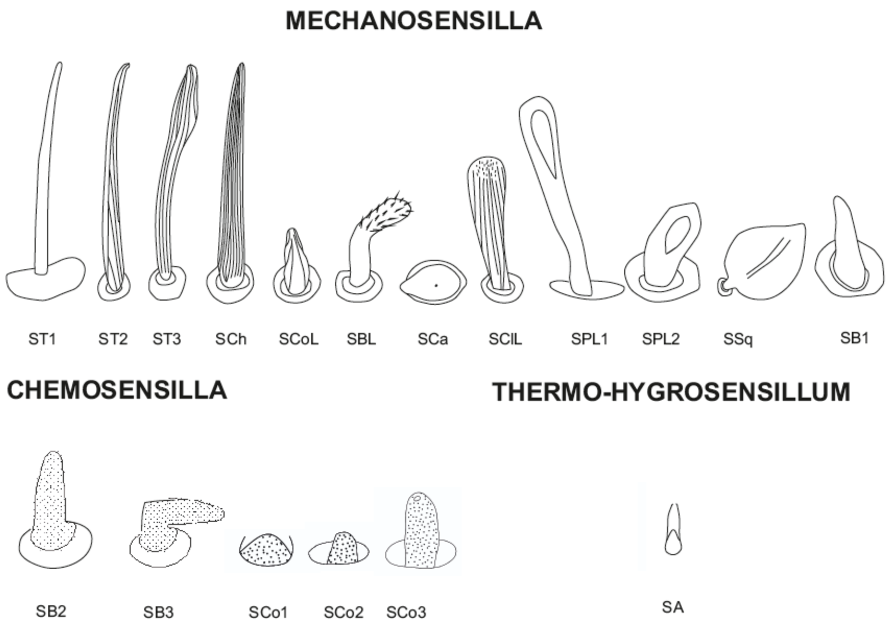

Figure S1. Types of the antennal sensilla in Nepoidea

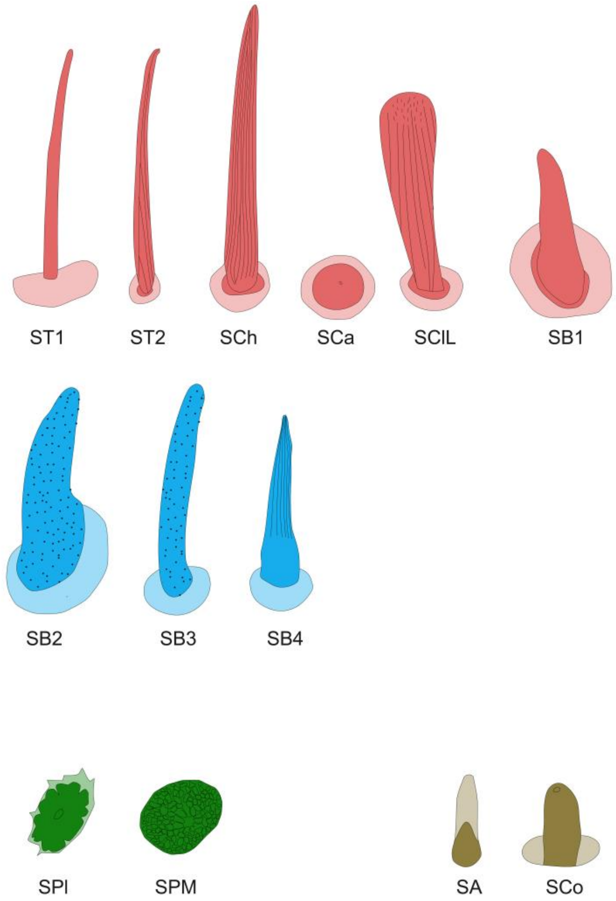

Figure S2. Types of the antennal sensilla in Ochteridae, Gelastocoridae and Aphelocheiridae.

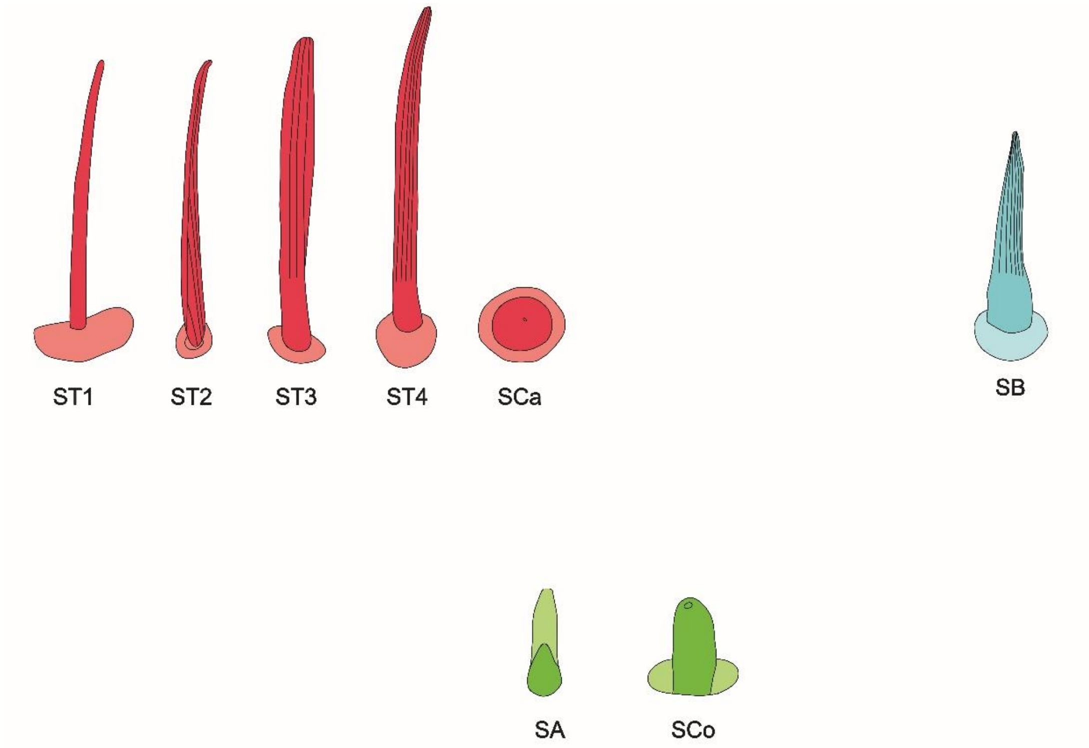

Figure S3. Types of the antennal sensilla in Corixoidea.

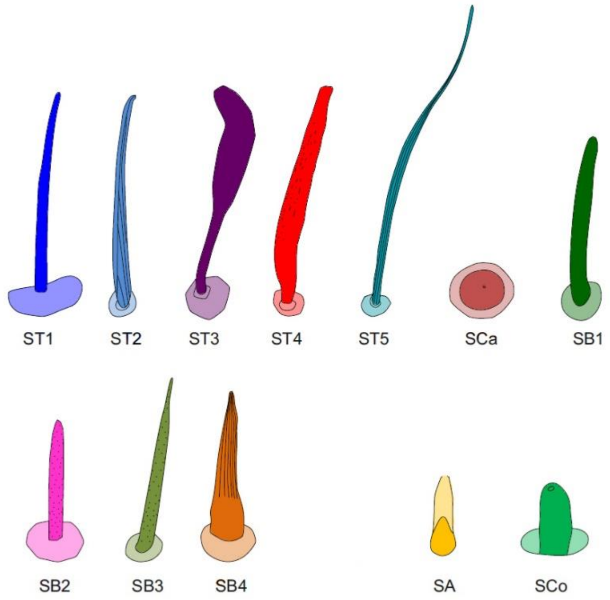

Figure S4. Types of the antennal sensilla in Naucoridae.
